# Designing an Adverse Drug Event Reporting System to Prevent Unintentional Reexposures to Harmful Drugs: Study Protocol for a Multiple Methods Design

**DOI:** 10.2196/resprot.5967

**Published:** 2016-08-18

**Authors:** David Peddie, Serena S Small, Katherin Badke, Maeve E Wickham, Chantelle Bailey, Adam Chruscicki, Christine Ackerley, Ellen Balka, Corinne M Hohl

**Affiliations:** ^1^ Centre for Clinical Epidemiology and Evaluation Vancouver Coastal Health Research Institute Vancouver, BC Canada; ^2^ School of Communication Simon Fraser University Burnaby, BC Canada; ^3^ Department of Emergency Medicine University of British Columbia Vancouver General Hospital Vancouver, BC Canada; ^4^ Department of Pharmaceutical Sciences Vancouver General Hospital Vancouver, BC Canada; ^5^ School of Medicine Queen's University Kingston, ON Canada

**Keywords:** adverse drug event, adverse drug reaction reporting systems, health services research, action research, qualitative research, user-centered design, methods, focus groups, systematic review

## Abstract

**Background:**

Adverse drug events (ADEs) are unintended and harmful events related to medication use. Up to 30% of serious ADEs recur within six months because culprit drugs are unintentionally represcribed and redispensed. Improving the electronic communication of ADE information between care providers, and across care settings, has the potential to reduce recurrent ADEs.

**Objective:**

We aim to describe the methods used to design Action ADE, a novel electronic ADE reporting system that can be leveraged to prevent unintentional reexposures to harmful drugs in British Columbia, Canada.

**Methods:**

To develop the new system, our team will use action research and participatory design, approaches that employ social scientific research methods and practitioner participation to generate insights into work settings and problem resolution. We will develop a systematic search strategy to review existing ADE reporting systems identified in academic and grey literature, and analyze the content of these systems to identify core data fields used to communicate ADE information. We will observe care providers in the emergency departments and on the wards of two urban tertiary hospitals and one urban community hospital, in one rural ambulatory care center, and in three community pharmacies in British Columbia, Canada. We will also conduct participatory workshops with providers to understand their needs and priorities related to communicating ADEs and preventing erroneous represcribing or redispensing of culprit medications. These methods will inform the iterative development of a preliminary paper-based reporting form, which we will then pilot test with providers in a real-world setting.

**Results:**

This is an ongoing project with results being published as analyses are completed. The systematic review has been completed; field observations, focus groups, and pilot testing of a preliminary paper-based design are ongoing. Results will inform the development of software that will enable clinically useful user-friendly documentation and communication of ADEs.

**Conclusions:**

We take this approach with the recognition that information technology-based solutions in health care often fall short of expectations as a result of designers’ failure to account for organizational and work practice considerations, and the needs of end-users. We describe how integrating qualitative methods into an iterative participatory design process (planned in partnership with end-users) will allow us to address specific clinical needs, conceptualize linkages between systems, integrate the reporting system into clinicians’ workflow, and design the system to optimize its uptake into practice.

## Introduction

Adverse drug events (ADEs) are unintended and harmful events associated with medication use, and represent a leading cause of ambulatory and emergency department visits and unplanned hospital admissions [1-4]. Prospective studies have consistently identified 30-70% of clinically significant ADEs as preventable [4-6], making the development, implementation, and evaluation of effective preventative strategies a public health priority [7]. To date the development of effective preventative strategies has been challenged by a lack of robust epidemiological data on patient and system-level risk factors for ADEs as well as the heterogeneity of clinical events observed. However, a prospective study enrolling a cohort of elderly patients admitted to hospital for an ADE indicated that a surprising 27% of patients were unintentionally reexposed to the culprit drug (the drug that caused the index ADE) within only six months of the initial event [8]. Similarly, Australian data indicate that up to 30% of ADEs requiring hospital admission consist of repeat events [9]. These data indicate that health systems interventions aimed at reducing repeat ADEs, regardless of their categorization or severity, may represent a high-yield area for prevention.

Poor documentation and lack of communication of ADE information between care providers, and across health care settings, are likely to contribute to frequent represcribing and redispensing of culprit drugs [10]. When examining health systems in which patients have multiple prescribers, it is clear that medical records are often fragmented and linkages between electronic resources remain inadequate. Crucial information regarding ADEs and high-risk medical conditions (eg, long QT syndrome) may remain elusive to care providers that prescribe and dispense drugs [10]. ADE documentation within electronic medical records (EMRs) has historically been limited to allergies, even though the majority of ADEs are not related to allergies (eg, medication-induced delirium, bleeding events) [3-4]. Most EMRs have not developed standardized, structured, user-friendly, and succinct data entry options for these types of events [11]. Even if information on ADEs is present within an EMR, it can be easily overlooked, as the information is often in lengthy free-text formats, buried within historical notes, and cannot be used to generate automated alerts that might remind the care provider of prior ADEs at the point of prescribing. Finally, these fields have not been configured to facilitate communication between care providers within, or across, health care settings (eg, between family physicians and community pharmacists).

Electronic ADE documentation currently occurs within online reporting systems hosted by organizations external to care delivery (eg, the United States Food and Drug Administration). Such reporting websites collect ADE data for post-market surveillance and research. Although this documentation is structured and somewhat standardized across systems, it is cumbersome and time-consuming to enter such information into existing systems. The resulting reports are used for research and regulatory purposes only, and cannot be accessed to support clinical care decisions or communication between health professionals [12]. ADE reporting within these websites is disconnected from the needs of clinical care providers, and clinicians rarely report such events, as immediate patient care-related activities supersede the data request of external agencies [13].

**Figure 1:**
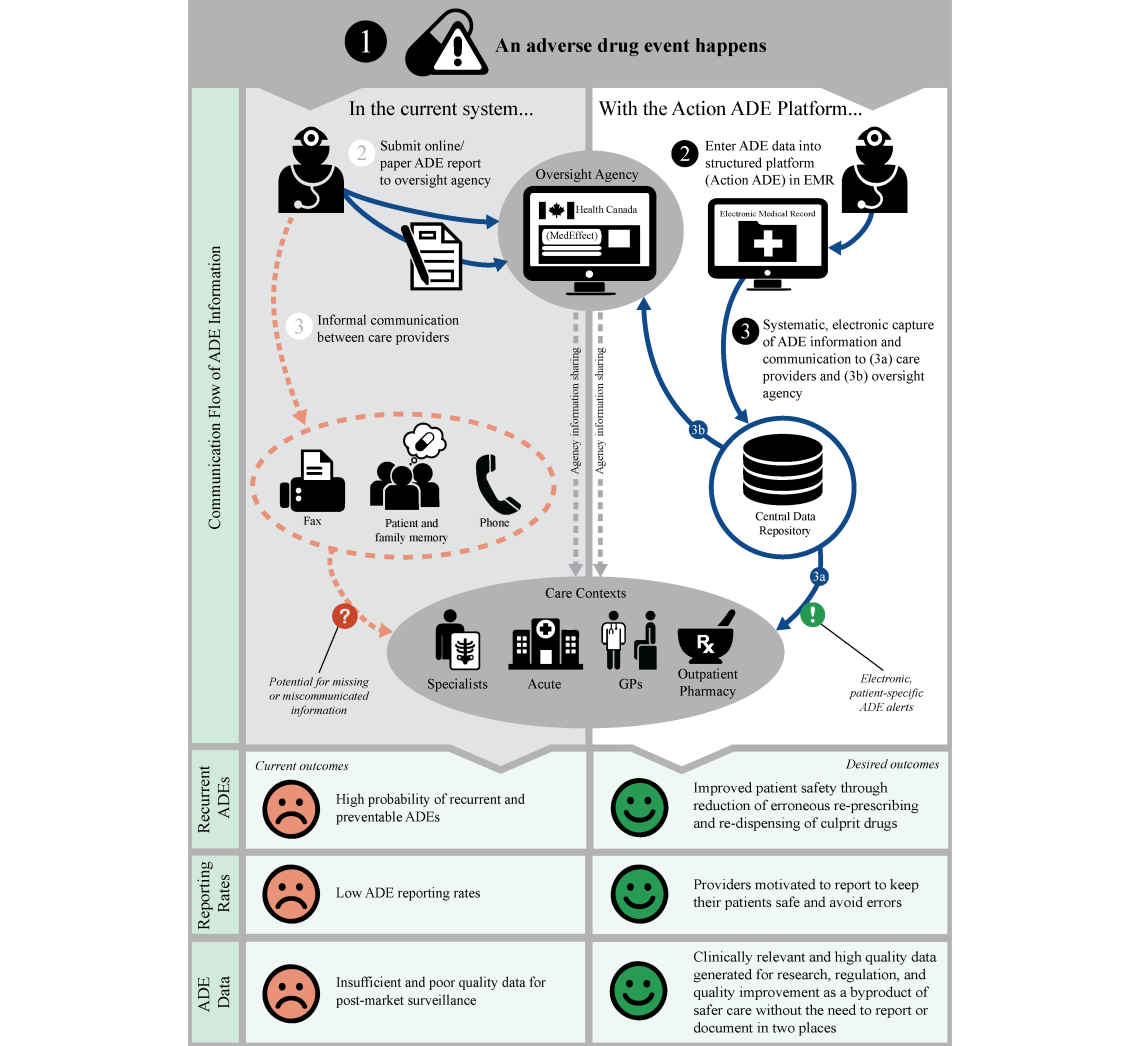
Adverse drug event information flow - existing versus with Action ADE.

If ADE reporting can be refocused to meet the documentation, communication, and patient safety needs of the clinicians who diagnose and treat ADEs at the point-of-care, and *hold* information about these events, clinicians may be more willing to document harmful events. Transforming the purpose of structured formal ADE reporting, from generating health data to improving clinical care by preventing recurrent events, may not only improve patient safety, but may yield *more* representative and higher quality reporting (and thereby generate more robust ADE data). Our main goal is therefore to design a patient-oriented and provider-centered ADE reporting system that is fully integrated into an EMR. Ideally, this system will be used by clinicians to facilitate ADE documentation and information flow between care providers and across health sectors (eg, between ambulatory care settings, hospitals, and community pharmacies) to prevent unintentional reexposures to harmful drugs (see Figure 1). A secondary goal is to use the structured ADE reports to generate improved health data on both known and novel ADEs for drug safety surveillance and research. We are not aiming to circumvent or replace the activities of pharmacosurveillance agencies, but rather to provide such agencies and other researchers with a novel source of rich ADE data. The objective of this paper is to describe the methods we plan to use to design the ADE reporting system, which we call *Action ADE*.

## Methods

### Approach

**Figure 2:**
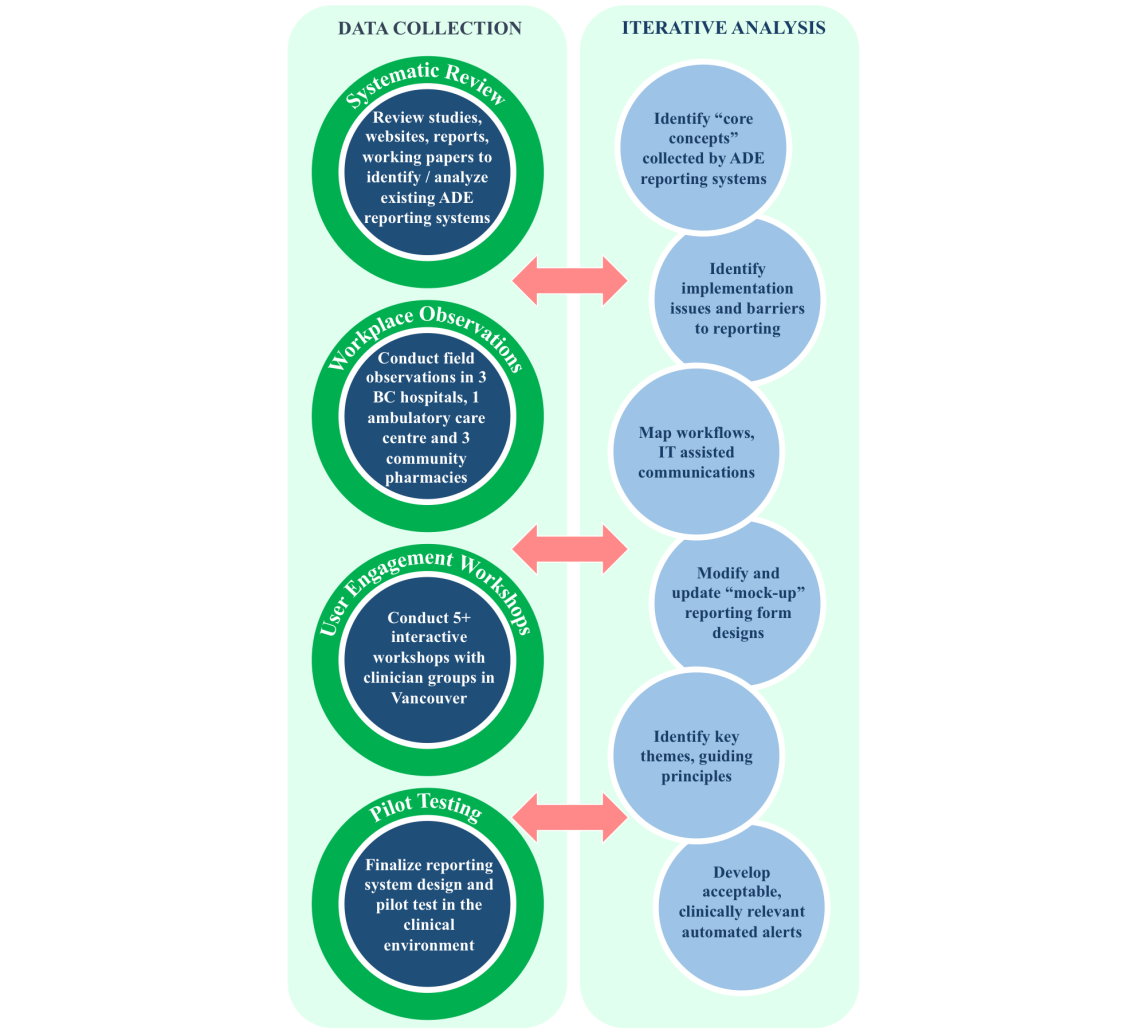
Planned data collection and analysis activities.

We will use action research and participatory design principles to develop a novel electronic ADE reporting interface. Action research involves teams of researchers and practitioners that incorporate various methods (eg, workplace observations, interviews, and focus groups) to integrate the perspectives of practitioners into innovative solutions for practical problems [14]. Incorporating principles of participatory design will allow us to ensure that our design reflects *observed actual* rather than *assumed* practices [15,16]. We anticipate that this approach will allow us to maximize the system’s user-friendliness, utility, and uptake into clinical practice. Figure 2 outlines the data collection and analysis activities that are planned for this study.

### Systematic Review

Our first step will be to complete a systematic review of existing ADE reporting systems [12,17]. Our goal is to identify how (and precisely which) ADE data are currently being solicited, and in what format and sequence ADE data are being collected. These findings will provide us with a foundation from which to propose a complete set of data fields for a future reporting system, to ensure that all relevant fields are considered.

We will begin by completing an environmental scan of the literature and developing a systematic search strategy. We will complement a bibliographic reference database search with a grey literature search, including a search of websites of pharmacovigilance organizations, to identify current ADE reporting systems internationally. The search will include qualitative and quantitative studies, government reports, working papers, and websites describing or hosting reporting systems for ADEs in humans. Reporting systems that focus only on errors or allergies will be excluded, along with registries that are specific to a single medication or class of medications, or a single disease state. Two authors will independently review all sources for inclusion and exclusion criteria, and then abstract data from all included systems. The extracted data will be exported into visual thinking software *Inspiration 9.2* to allow us to visualize and sort information pertaining to the data fields, structure, and dictionaries used by each system. When using *Inspiration 9.2*, each extracted data field will appear as a single bubble containing the label of the individual data field within the reporting system, and the number of times we encountered the same data field across systems. We will then sort the individual data fields into overarching reporting concepts and, in a second iteration, all duplicate (or very similar) data fields will be eliminated. During a third sort, relationships and hierarchies between the reporting fields and concepts will be identified.

This process will allow us to distill a compendium of data fields from individual reporting systems into a list of *core fields* which are currently used to communicate ADE content in existing reporting systems. We will then develop a preliminary ADE reporting form, using all core fields that we have identified.

### Observational Fieldwork

In parallel to our systematic review, we will conduct observational fieldwork to observe physicians and pharmacists in settings with patients who commonly experience ADEs. This approach will allow us to gain an understanding of clinical workflow, the work environment, how ADEs are diagnosed, documented, and become reportable, and barriers to reporting. Furthermore, this approach is useful for understanding users’ actual needs, and the context in which the technology will be integrated, rather than relying on intuition and untested assumptions [18-20]. For example, we might gain insight into the collaborative nature of ADE diagnosis, make tacit elements of work explicit, and show the differences between how work is conceptualized as opposed to how it actually unfolds in real-life (eg, using spontaneous problem-solving processes) [15]. In addition, our fieldwork will allow us to observe the roles of relevant actors and artefacts that might otherwise go unnoticed and not be considered in our design, especially those resources used to gather and document information that contributes to medication management decisions.

Our team will conduct observations in the emergency departments and on the wards of two urban tertiary hospitals and one urban community hospital, in one rural ambulatory care center, and in three community pharmacies in British Columbia, Canada. Researchers will shadow clinical pharmacists, nurses, and physicians at various times of the day (and days of the week) to account for changing levels of activity and work procedures over time. Study participants will include a convenience sample of practitioners recruited through the contacts of the practicing clinicians on our team, and those on shift at the time of scheduled data collection shifts. Our observations will focus on (1) the setting, (2) patient presentations in which ADEs are suspected, managed, and documented, (3) artifacts that mediate the work (eg, medication reconciliation forms), (4) activities that constitute work (eg, obtaining a medication history *),* and (5) information flow between clinicians. Two researchers will independently code observation notes using the qualitative data analysis software *NVivo 10*, and iteratively review the data with attention to emerging trends and concepts. Upon initial review, our team will develop a formal coding structure while ensuring consistency between coders. As coding progresses, we anticipate the need to discuss emerging findings in team meetings, and will use analytic exercises, including situational maps (a set of visual thinking exercises for interrogating qualitative data [21]), workflow diagrams, information flow maps, and event summaries to explore our findings.

### Participatory Workshops

Our team will synthesize information from our systematic review and field observations to create a preliminary ADE reporting form. This form will take into account all relevant data fields identified in our systematic review, the sequence in which documentation can best be adapted to the workflow observed in our fieldwork, and required linkages to other information sources. We will generate the preliminary form using *Microsoft Visual Basic for Applications* so that it has the appearance of a computer screenshot. This template will enable clinicians to imagine how the reporting interface might look. The first iteration of the form will be a *maximum* form containing data fields that reflect all core reporting concepts that were encountered in our systematic review. We will use this template as a starting point to engage with clinician end-users, in order to obtain feedback and refine the form.

We will conduct a minimum of five participatory workshops with different clinician groups (emergency physicians, family physicians, hospitalists or internists, hospital pharmacists, and community pharmacists) in which we will present ADE cases along with the preliminary ADE form, in order to stimulate discussion. These workshops will create opportunities for bottom-up collaborative design, in which future end-users can choose between design alternatives, highlight perceived challenges and opportunities, propose solutions, and point to issues that we may not have identified. Unlike observational or interview settings, workshops will allow participants to develop their views in concert with their peers through discussion, challenging their personal assumptions and introducing perspectives that they may not have previously considered [22].

We will hold workshops during lunchtime professional rounds for groups practicing in hospitals, and advertise via posters and email invitations. For participants practicing outside of hospitals, we will hold workshops in the evenings and recruit clinicians through verbal communication, posters, and email invitations. Each workshop will begin with an informal survey of the participants to gather information on their practice setting, the information systems they use, and the participants’ concerns related to ADE documentation and communication. Using the forms and a wide range of ADE cases from our observations as examples, we will ask participants to highlight the data they feel must be documented, and where and how it should be documented. We will note aspects of the form that they find useful or problematic, and ideas about how the form might be improved. After each workshop, we will revise the ADE form to incorporate the end-users’ feedback, in order to create the next version of the form that will be presented in the following workshop. We will create a log of changes to the form, including a rationale for each change. Workshops will continue until the form is acceptable to the groups involved, and no novel suggestions or concerns are being raised.

### Paper-Based Pilot Testing

Once a mature paper-based design is established, we will pilot the form in the clinical setting prior to building it in electronic format. Our main goal is to test the content, functionality, and clarity of the form. We will observe how reporters access information sources while completing the form to understand the links between our form and other electronic systems. This approach will allow us to address required revisions that could not be anticipated during the workshops, and consider linkages between systems that must be established prior to the electronic build. We anticipate that paper-based pilot testing will allow us to propose a more mature design, and introduce revisions at lower cost, than if revisions were required after all programming costs have been incurred.

We will conduct semi-structured interviews and use *lightweight ethnography* (a methodology that allows for collection of specific and relevant information, while accepting that a complete understanding of a work setting may not be needed [23]) to observe clinicians completing the forms. This approach is ideally suited for pilot studies, as it can rapidly and efficiently provide guidelines for technology design.

Study participants will consist of a convenience sample of clinical pharmacists that will be recruited through team contacts, specifically via verbal communication and email invitations. During 2-4 hour shifts, a research assistant will shadow participants in two hospital settings that commonly encounter patients with ADEs. The research assistant will provide pharmacists with a paper version of the ADE reporting form and ask them to complete it should they encounter an ADE. The research assistant will observe the participants completing the form, and collect additional information about the ADE, workflow, and any comments and impressions about the reporting form. Coding and analysis of qualitative data from field notes will be conducted in the same manner as the observational fieldwork. We will produce descriptive statistics to summarize the completeness of data entry regarding individual data fields and the form as a whole.

### Development of Automated Alerts

Once a paper-based ADE reporting form has been established, with a robust set of data fields making up the *input* into the reporting system, we will develop our system’s *output*. Output screenshots are those through which an ADE will be flagged when attempts are made to represcribe or redispense a culprit drug (or drug of the same class). The output will be integrated into the patients’ EMR, and communicated to and stored in PharmaNet, British Columbia’s electronic outpatient drug dispensing database. By storing alerts in patients’ PharmaNet profiles, we will be able to communicate alerts to care providers (with access to PharmaNet) who practice outside of the institution in which the ADE was diagnosed. Such providers include family physicians, who usually practice in offices without access to hospital EMRs, as well as community pharmacists who must enter dispensed drugs into PharmaNet for reimbursement purposes.

Alert functions can be valuable tools in health care. However, when alerts are too frequent, not specific enough (eg, flagging many clinically irrelevant events), or integrated poorly into clinical information systems, they may impede and complicate clinical work, or contribute to alert fatigue. Alert fatigue occurs when clinicians are bombarded with irrelevant or obvious alerts that they learn to bypass, override, or ignore [24]. Thus, we will avoid generic alerts that are not specific to individual patients (eg, drug interactions) by generating alerts for serious and confirmed ADEs that will only be flagged for the specific patient in whom the ADE was reported, and only when the culprit drug or drug class is represcribed or redispensed.

To ensure that ADE alerts communicate the correct information in a timely and appropriate manner, we will use workplace observations and participatory workshops (as previously described) with an emphasis on providers in outpatient settings, who would receive ADE alert outputs (eg, family physicians and community pharmacists). The same methods will be used for recruitment, scheduling of data collection shifts, and analyses, and will focus on the content and display of ADE information, and when/how alerts should be integrated into clinical workflow. Using the ADE data elements from our ADE reporting form (ie, the input), and our workplace observations, we will design preliminary ADE alerts (ie, the output). Participatory workshops will facilitate the creation of preliminary ADE alerts, and participants will highlight the data that they feel is required, along with any display modification suggestions or issues with missing information. We will note any ideas that might improve the alerts, and after each workshop we will revise the ADE alerts to incorporate the end-users’ feedback, in order to optimize the form that will be presented in the following workshop. Again, workshops will be held until the ADE alerts are acceptable to the groups involved, and no novel suggestions or concerns are being raised.

## Results

This is an ongoing project, with results being published as analyses are completed. The systematic review of existing ADE reporting systems has been completed and published [12]. Further data collection (field observations, focus groups, and pilot-testing) has begun, preliminary analyses are underway, and results are to be expected in 2016-2017. These results will inform the design of a clinically useful and user-friendly platform for communicating ADEs. The platform will be systematically evaluated beginning in 2017.

## Discussion

### Study Rationale

Existing ADE reporting systems have been designed at a distance from use, with limited clinician end-user input. Current systems have focused on the data needs of organizations engaged in medication safety surveillance, rather than the information needs of clinical care providers who diagnose and treat patients with ADEs. Research from multiple jurisdictions has consistently revealed that clinicians are simply not using these systems, as the act of reporting is perceived as irrelevant to clinical care delivery, and systems are cumbersome to use and not integrated into current electronic information systems [25]. As a result, the vast majority of ADEs (even serious events) remain unreported, and are not reflected in current health data that are used by pharmacovigilance and research organizations that examine drug safety. Preventable ADEs go unaddressed, to the detriment of patients, the health care system, and taxpayers.

Our work addresses a methodological gap in the way that ADE reporting systems (and other health information technology systems) have been conceptualized, designed, and implemented. Given the recognized limitations of the current state of ADE reporting, innovations have been proposed by others. However, these innovations preserve the data-centric orientation of the current system. Some studies, for example, have suggested using administrative data or mining EMRs for signals to identify harmful events [26,27], although validation studies have indicated that these methods have poor sensitivity for identifying relevant ADE outcomes [28,29]. Other studies have pointed to the value of expanding the role of patient reports, yet there has been little formal evaluation of patient reporting, and reporting rates among patients remain low [30,31]. Studies that have examined underreporting and initiatives to improve reporting have rarely scrutinized systems issues, instead focusing on the users. The results of these studies attribute shortcomings to poor user knowledge, attitudes, workplace culture, professional priorities, incentives, and media influence [13,32]. Studies have generally failed to question the current data-centric orientation of reporting systems, examine system shortcomings, or propose ways to redesign reporting so that it may complement and facilitate components of clinical care, in addition to meeting the data needs of external organizations.

In contrast to previous studies and prior interventions that attempted to address underreporting, we suggest that it is not the end-users that need to be *fixed* through more education, enticement, or enforcement, but rather that the work practices and technologies that support their work need to be altered. The conscious work of end-users cannot be replaced by analyzing traces of data left as documentation within medical records that were never specifically intended to capture robust ADE information [28,29]. Instead, we stress the need to rethink the rationale and systems designed for reporting ADEs. If clinicians are going to supply the information-reporting that systems seek to capture, we must prioritize the design of such systems so that they work for clinicians, enabling them to meet their care delivery goals of safer and more efficient care for patients.

Medical disciplines have generated little or no discussion about what, if any, research evidence should be used to inform the design of information technology systems that may have a profound impact on health services delivery. The use of qualitative observational data (including information from end-user engagement) in designing information technologies has gained popularity in the last 25 years in computer and information sciences [33]. Despite the promise that such approaches hold, their uptake in medical communities has been slow, and is likely related to assumptions that frame the design of health information technologies as exclusively a *technical* problem [19,34,35]. The claims for technologies such as EMRs, automated decision support, or computerized physician order entry–that they will increase efficiency, support decision-making, reduce errors, and standardize information–are often taken as self-evident [19]. The benefits are simply presumed to follow logically from implementation, and coupled with this assumption is the notion that staff and clinical practices will *adapt* to new technology [34].

In practice, however, the success of a new technology often hinges on how well it is integrated into organizational and clinical practice, and whether it meets the needs of end-users [19,34,35]. In order to optimize user-friendliness, functionality, and uptake, methods are needed to bring rigor, robustness, and accountability to this process. Importantly, these methods must allow for meaningful engagement with clinician-users in the design, evaluation, and implementation phases, and should include observational methods to identify differences between actual and perceived work practices. The methods we outline in this paper offer an example of how qualitative research methods may be integrated in an iterative fashion to meet this need. Elements of a systematic review can be used to ensure that an information technology design begins from a complete account of systems in existence (including non-electronic formats). From this starting point, designers may choose from a variety of observational and participatory design methods to generate further evidence to inform system design. This approach may be used to reinvent existing information systems that, over time, have become part of a *de facto* and perhaps antiquated infrastructure [36].

### Limitations

Careful methodical design does not guarantee uptake into clinical practice. Therefore, our work must continue after the intervention has been introduced, and include evaluation and refinement of the design, and knowledge dissemination to end-users. To foster successful uptake, we must first support the design stages with training, education, program evaluation, and refinement of the interface. Second, the introduction of any reporting system will be perceived as an additional burden of documentation on clinicians, whose priority remains patient care. Accordingly, the introduction and adaptation to a new electronic documentation platform is likely to be met with some resistance, and any added documentation must be minimized. We hope that our collaborative approach will help mitigate this resistance by making this documentation practical for clinicians and their patients, and help clinicians meet patient safety goals. Third, shifting communication between practitioners regarding ADEs to an automated platform carries the risk of reducing verbal communication. While verbal communication about ADEs is presently inadequate, we need to be attentive to how an intervention might negatively affect collaborative practice to the extent that it currently exists. Fourth, the dissemination of unverified ADE information among care providers introduces a number of concerns, such as data reliability (eg, false positives), patient privacy, and provider liability. These serious issues must be evaluated during and after implementation, along with our assumptions that the *Action ADE* system might improve reporting rates, prevent recurrent events, and provide higher-quality data.

These concerns will be the foci of future phases of our work that will be devoted to the implementation and evaluation of the system. Finally, our research will be undertaken in the health care environment in British Columbia, Canada, with specific provider groups, and will be influenced by specific contexts that will be encountered. Successful integration of this tool into other settings will require consideration of (and adaptation to) local exigencies and concerns, will require collaboration with teams in other settings, and involve tailored education and implementation. To address these issues, we plan to carry out work in additional communities of practice to ensure that our design is relevant across multiple health care environments.

### Conclusion

In this paper, we have outlined an action research and participatory approach to designing a novel, provider-centric reporting tool to capture and share information about ADEs experienced by patients, in order to reduce the likelihood of ADE recurrence. This approach may be useful in enhancing patient safety, while generating robust and representative data on ADE outcomes for drug safety and effectiveness research and regulation. As policies and practices shift to accommodate new federal laws that mandate the reporting of all serious adverse drug reactions in Canada, our work may offer a model for how technological innovation in health care systems design can be planned in partnership with health providers.

## Abbreviations

ADE: adverse drug event
EMR: electronic medical record
